# Study on the Microstructure Evolution and Tungsten Content Optimization of 9Cr-3W-3Co Steel

**DOI:** 10.3390/ma11112080

**Published:** 2018-10-24

**Authors:** Longteng Ma, Yanfeng Wang, Guobiao Di

**Affiliations:** Shougang Research Institute of Technology, Beijing 100043, China; wangyf@shougang.com.cn (Y.W.); abiaosky@163.com (G.D.)

**Keywords:** tungsten, creep rupture life, lath substructure recovery, precipitation coarsening

## Abstract

Creep rupture tests of 9Cr-3W-3Co steel were conducted in the range of 120 to 200 MPa at 650 °C. The influence of stress on microstructure evolution was investigated in detail. In the high stress regime, a large density of dislocation was generated and induced precipitation of fine and dispersive particles. However, at lower stresses, a transformation from martensite laths to large size subgrains and a coarsening of precipitates took place due to significant recovery and loss of pinning effect during long term exposure. Thermodynamic results revealed decreasing tungsten content effectively retarded the coarsening behavior of M_23_C_6_ and Laves phase, hence further improvement of creep rupture time was achieved experimentally.

## 1. Introduction

In the ultra-supercritical (USC) coal-fired power plants, 9–12% Cr heat resistant steels are widely used as structural materials for boilers, main steam pipes and tubes [1]. The superior creep strength of 9–12% Cr steels is due to the high microstructure stability at elevated temperatures [2]. The substructure of these steels consists of prior austenite grains (PAGs), tempered martensite laths, high-density dislocations and dispersive second-phase particles. Typical second-phase particles in 9–12% steels are (Nb,V)-rich MX carbonitrides, Cr-rich M_23_C_6_ carbides and Fe_2_(Mo,W) Laves phase [3,4]. MX particles are mainly formed within grains during tempering, while M_23_C_6_ and Laves are mostly precipitated along grain or subgrain boundaries during subsequent aging or creep. The key factor that affects the microstructure evolution is the interaction between precipitates and other substructures. In the case of no transformation to Z phase, MX particles exhibit great coarsening-resistance and the pinning effect on dislocations by MX could be well maintained during high temperature exposure. At the initial stage of creep, fine and dispersive M_23_C_6_ and Laves phase particles contribute high creep-resistance by exerting great Zener drag force on subgrain boundaries, whereas the high growing rate of these precipitates degrades this effect gradually [5,6,7]. Recent works [8,9,10] suggest that the coarsening behavior of M_23_C_6_ could be effectively suppressed by the optimized addition of boron, which segregates near boundaries and decreases the interfacial energy between matrix and M_23_C_6_. Early works reported that eliminating silicon slows down the coarsening process of Fe_2_Mo Laves phase in 9Cr-2Mo steel [11,12] but little research concerning the method to slow down this coarsening rate of Fe_2_W Laves phase has been reported.

Traditional martensitic steels such as T/P92 and T/P122 cannot meet the requirement of long-term service at temperatures higher than 600 °C, while a novel 9% Cr steel, 9Cr-2W-3Co, with superior creep strength was developed in recent decades.

Much has been reported on alloy design, heat treatment optimization and structure evolution during aging [13,14,15,16]. However, little attention has been paid to the influence of stress on substructure recovery and precipitation behavior in 9Cr-3W-3Co during creep. Although the creep-rupture strength of 9Cr-3W-3Co steel is much higher than that of any other 9–12% Cr steel, further improvement of creep resistance is still imperative. The author reported the Laves phase evolution during long-term stress-free expose using chemical phase analysis method [17]. In the present study, the effects of varying stress on the evolution of the microstructure of 9Cr-3W-3Co steel was investigated in detail, followed by the coarsening kinetics of precipitates through thermodynamic simulation, while a method for further improvement of creep property was also developed.

## 2. Materials and Methods

### 2.1. Materials and Heat Treatments

**Figure 1:**
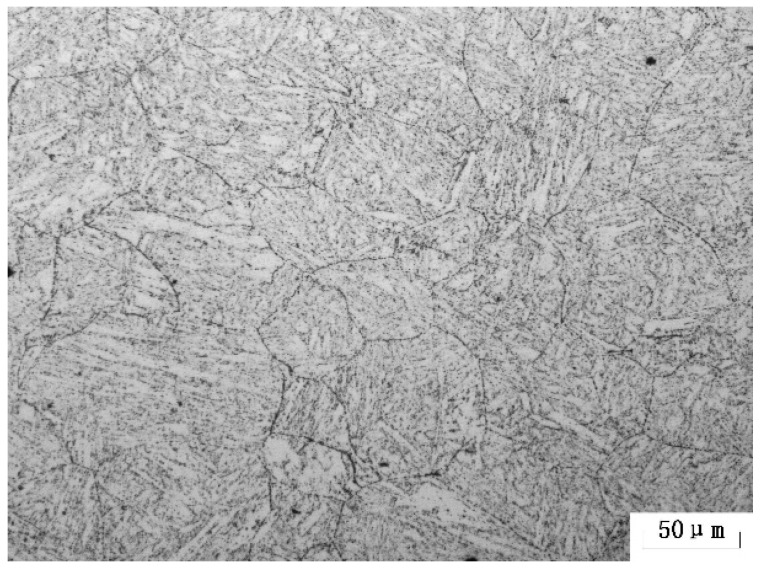
Optical microscopy of 9Cr-3W-3Co steel in the original condition (normalized and tempered).

All the 9Cr-3W-3Co steels, with the chemical composition listed in Table 1 were melted in a vacuum furnace. The tungsten content of these steels ranges from 2.36 to 2.93%. After being melted, 50kg ingots were homogenized at 1150 °C, then air-cooled to room temperature. After that, the steels were forged into round bars with a diameter of 14 mm at 950–1150 °C. Specimens with a length of 80 mm were cut longitudinally from these bars for heat treatment. Heat treatment, with normalizing at 1100 °C for 1 h followed by air-cooling and tempering at 780 °C for 3 h with subsequent air-cooling, was performed on all experimental specimens. Since the content of W in N# steel is 2.96%, or close to 3%, N# steel was the first to undergo creep tests, followed by the other 2 steels. In this investigation, specimens for microstructure observation were all from N# steels. The original microstructure of 9Cr-3W-3Co steel is tempered martensite, as shown in Figure 1.

### 2.2. Creep Tests and Microstructure Observations

**Figure 2:**
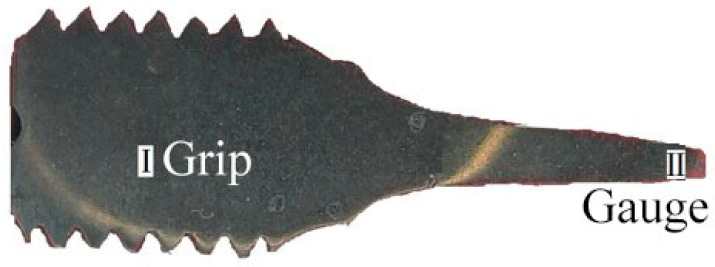
Slice from the ruptured sample.

At 650 °C, creep tests under constant loads were carried out on these steels in the stress range of 100–200 MPa. Cylindrical specimens with a gauge diameter of 5 mm and a gauge length of 25 mm were used in all the creep tests. For convenience sake, a slice with a thickness of 1.5 mm was longitudinally split from the ruptured sample. Each slice consisted of a grip and a gauge, which were marked section I and section II, respectively, as shown in Figure 2.

These slices for field emission scanning electron microscopy (FESEM) and electron back scattered diffraction (EBSD) analysis were then prepared using mechanical and electrolytic polishing. The SEM and EBSD tests were carried out using a JEOL 7200F microscope (JEOL, Ltd., Tokyo, Japan) equipped with an Oxford electron back-scattered diffraction detector. Thin films for transmission electron microscopy (TEM) specimens were prepared using mechanical milling and conventional dual-jet electro polishing in a solution of HClO_4_ + CH_3_Ch_2_OH at a temperature of −25 °C, then further thinned by ion beam milling. TEM was performed using a Hitachi H800 microscope (Hitachi High-Technologies Corporation, Tokyo, Japan).

## 3. Results

### 3.1. Precipitation Evolution of 9Cr-3W-3Co Steel

**Figure 3:**
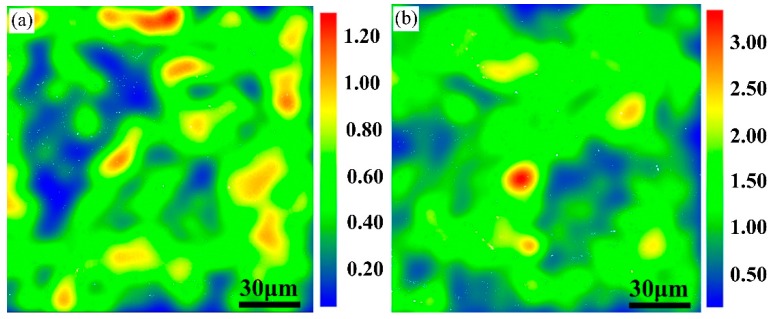
Strain contouring maps of N# steel ruptured sample (**a**) grip; (**b**) gauge.

To estimate the extent of deformation, strain contouring maps were acquired from EBSD data to exhibit the stress condition of grip and gauge sections cut from the same sample [18]. The strain contouring maps of the ruptured sample (N# steel) are given as an example in Figure 3.

**Figure 4:**
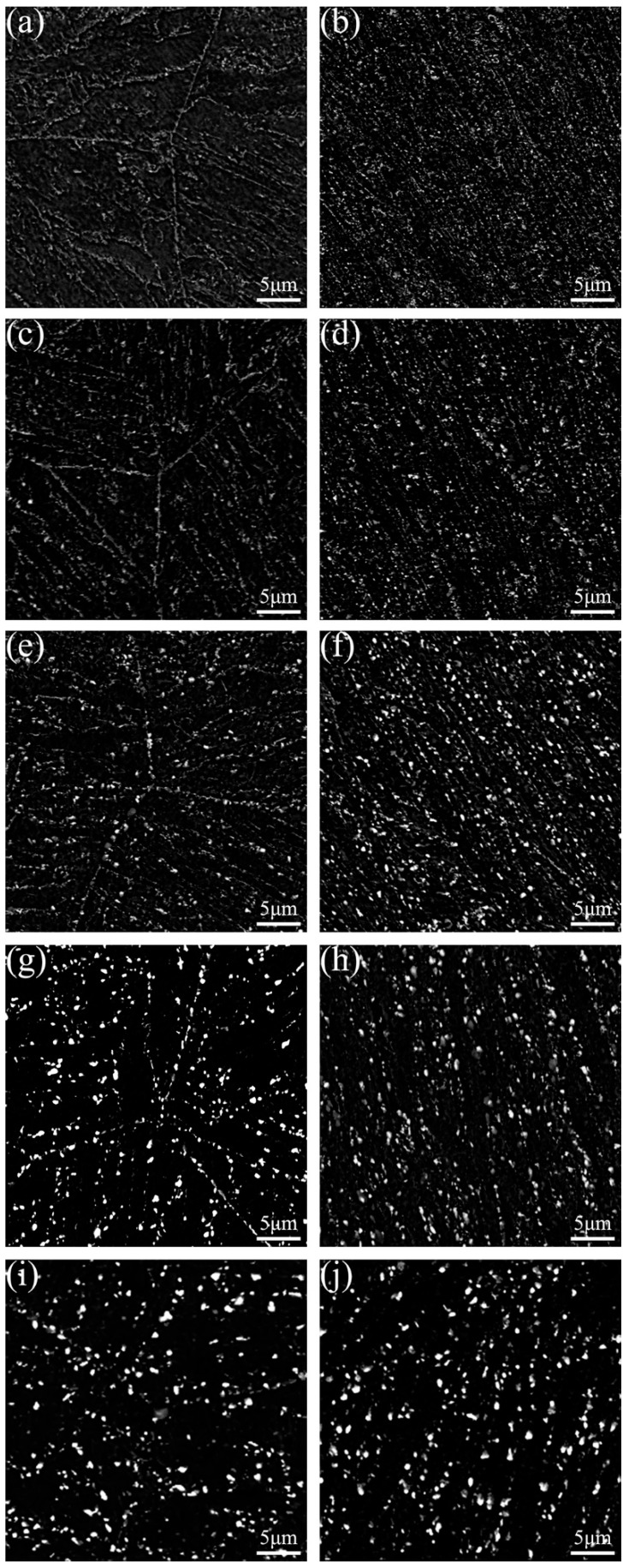
SEM images of 9Cr-3W-3Co steel after creep tests at 650 °C: specimens ruptured after (**a**,**b**) 134 h, (**c**,**d**) 837 h, (**e**,**f**) 3037 h, (**g**,**h**) 6667 h, (**i**,**j**) 17,991 h. (**a**,**c**,**e**,**g**,**i**) are from grip areas; (**b**,**d**,**f**,**h**,**j**) are from gauge areas.

As shown in Figure 3, the grip section was approximately stress-free, which was similar to samples after aging, while the gauge area was under a relatively high strain level. For the gauge area, high temperature and strain would exert a cooperative influence on the microstructure evolution, which is quite discrepant with the rest of the creep specimen. For 9Cr-3W-3Co steel, Cr-rich M_23_C_6_ and Nb/V carbonitrides are precipitated during tempering, while W-rich Laves is precipitated during long-term creep [19]. Besides, it has been reported that the dominant factor that brings about creep failure is the coarsening behavior of Laves phase [20]. Thus, to uniquely distinguish W-rich Laves phase, typical microstructures that evolved in the grip and gauge sections of specimens subjected to creep at 650 °C under various stresses in the range of 120 to 200 MPa are shown in Figure 4.

**Figure 5:**
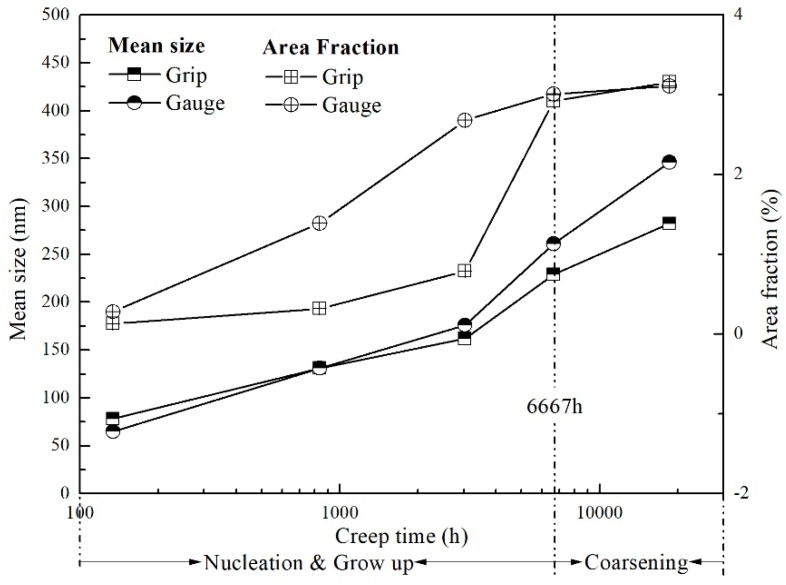
Quantitative analysis of Laves phase evolution during creep.

Apart from the coarsening behavior of Laves phase commonly occurring in both grip and gauge, the precipitates are distributed in two ways. In the grip area, Laves phase particles were distributed uniformly around grain and sub-grain boundaries, while these precipitates could be easily found along the tensile direction in the gauge section. The area fraction and mean size of Laves phase in these two areas were compared, as shown in Figure 5. A critical rupture time that divided the whole process into two-stages was 6667 h. After short-term creep, the mean size of Laves phase in these two areas was close, while the area fraction of this phase in the gauge area was much larger than in the grip area, indicating that stress is quite helpful for the nucleation of Laves phase. However, after long-term creep, the larger size of particles in the gauge area revealed the accelerated coarsening behavior induced by the stress.

### 3.2. Lath Substructure Evolution of 9Cr-3W-3Co Steel

**Figure 6:**
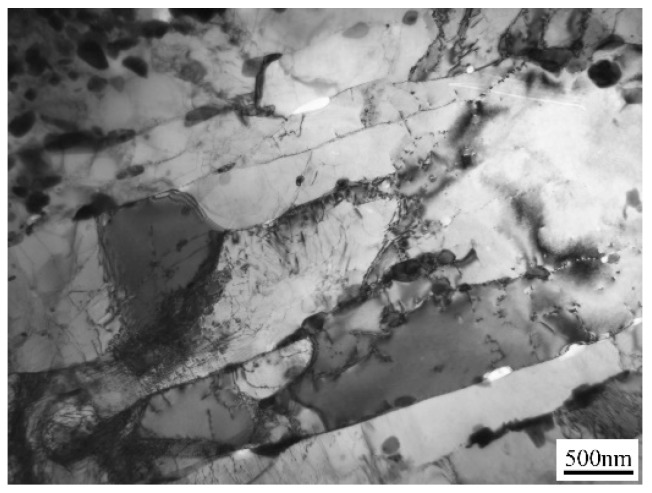
TEM image of the original microstructure of 9Cr-3W-3Co steel.

**Figure 7:**
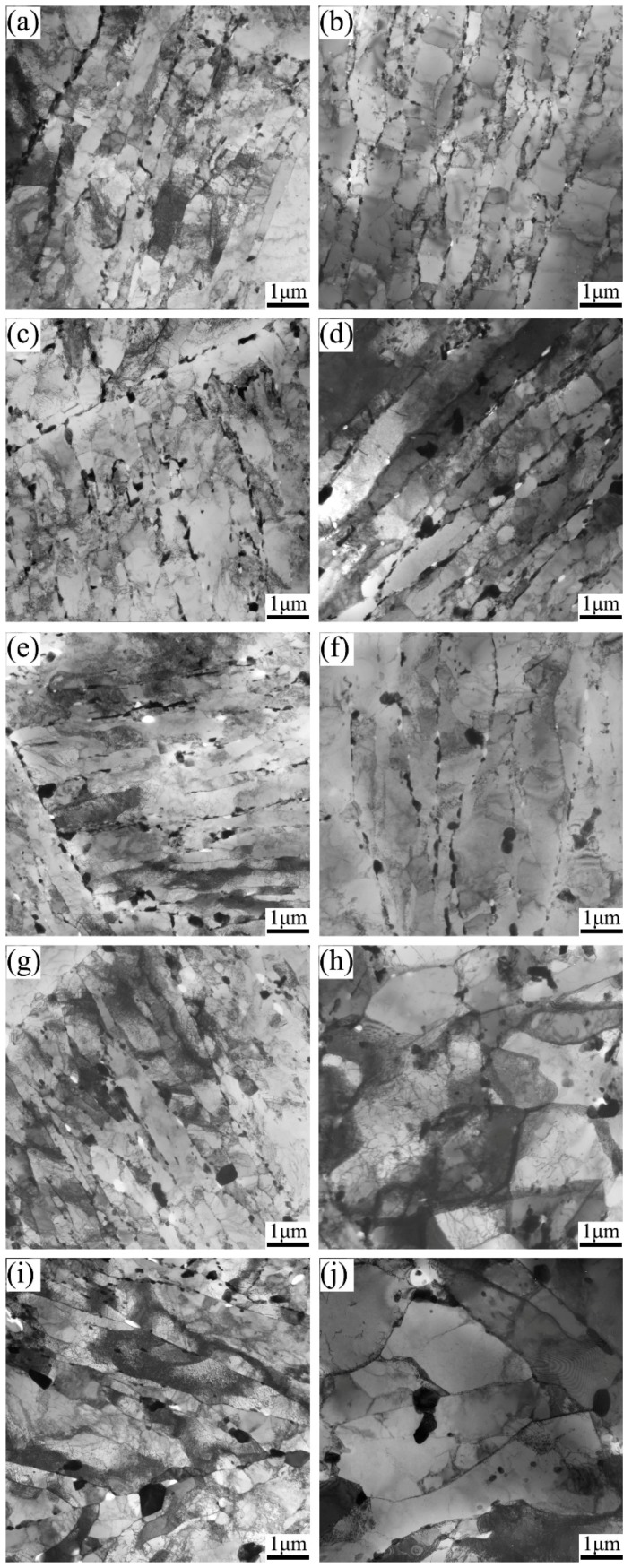
TEM images of 9Cr-3W-3Co steel after creep tests at 650 °C: specimens ruptured after (**a**,**b**) 134 h, (**c**,**d**) 837 h, (**e**,**f**) 3037 h, (**g**,**h**) 6667 h, (**i**,**j**) 17,991 h. (**a**,**c**,**e**,**g**,**i**) are from grip areas; (**b**,**d**,**f**,**h**,**j**) are from gauge areas.

**Figure 8:**
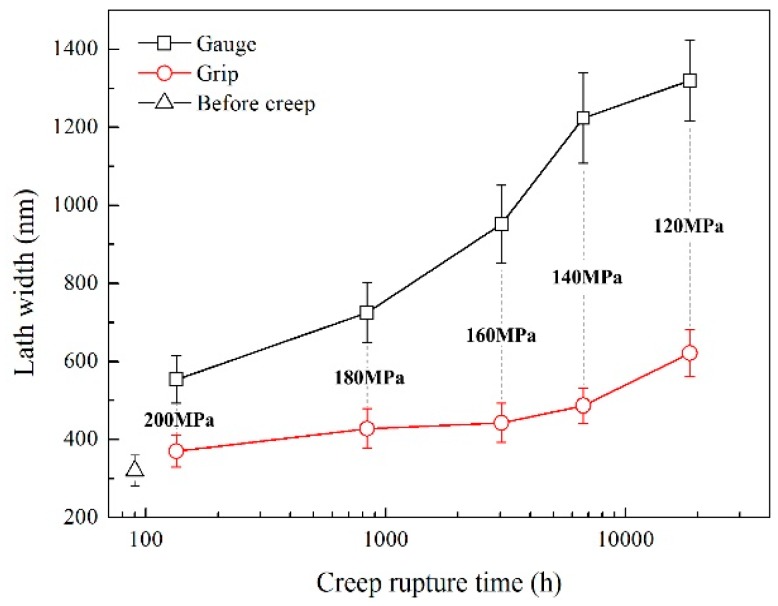
Lath width of 9Cr-3W-3Co creep ruptured samples.

TEM images are given in Figure 6 and Figure 7 to show the matrix evolution of 9Cr-3W-3Co steel under different creep stress. The lath substructure in the grip area could be maintained during long-term creep, while intensified recovery was found in the gauge sections. Particularly, for specimens under stress lower than 140 MPa, the substructure transformed from lath into subgrains. The width of the substructure was measured and the result is shown in Figure 8. For specimens in the grip area, there was no significant variation in magnitude of lath size due to the different creep rupture time. However, the magnitude of lath width in the gauge section was substantially enhanced, while the final size changed from nanoscale to over 1.3 μm. This reveals the significant role of stress in the recovery process.

## 4. Discussion

### 4.1. Dislocations, Accelerated Coarsening and Recovery Process

**Figure 9:**
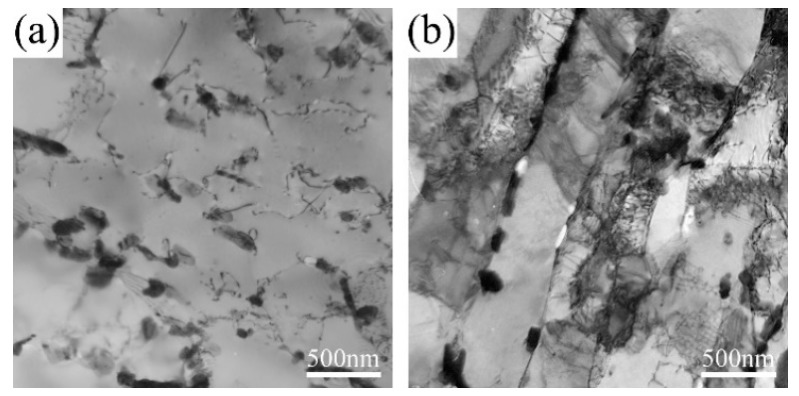
TEM micrographs of 9Cr-3W-3CO creep rupture specimens under stress of 200 MPa: (**a**) gauge, (**b**) grip.

During the short-term creep tests, a high density of dislocations induced by stress formed at the gauge sections. Also, there was a large amount of Laves phase particles related to the ends of dislocation lines, as shown in Figure 9. This suggests that the number of Laves phase nuclei could be significantly improved; hence, the dispersed distribution could be attributed to the stress states. In contrast, the Laves phase particles in grip sections were found near lath boundaries, which is similar to that under aging treatments. Therefore, the stress state is the substantial factor that influences the distribution of Laves phase.

**Figure 10:**
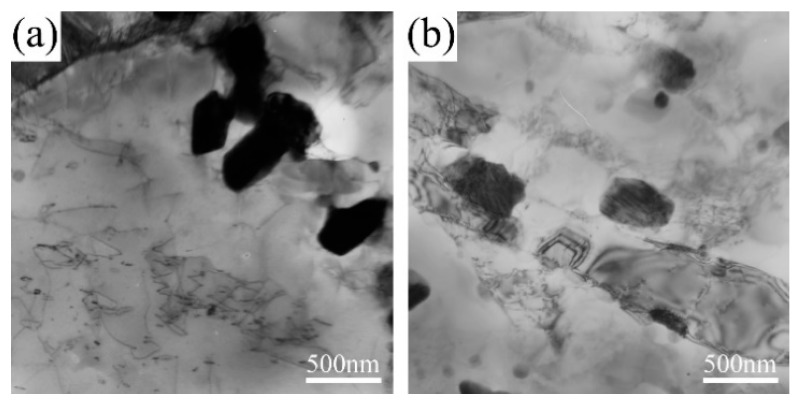
TEM micrographs of 9Cr-3W-3Co creep rupture specimens under stress of 120 MPa: (**a**) gauge, (**b**) grip.

The time to rupture for 9Cr-3W-3Co steel under low stress was 6667 h for 140 MPa and 18,551 h at 120 MPa. For specimens under these stresses, the area fraction of Laves phase particles was very close, which indicates that coarsening is the dominant mechanism. Although the movement and annihilation reduced the large number of dislocations during long-term creep, the energy provided by the stress quickened the coarsening process of precipitates at the gauge sections, as shown in Figure 10.

**Figure 11:**
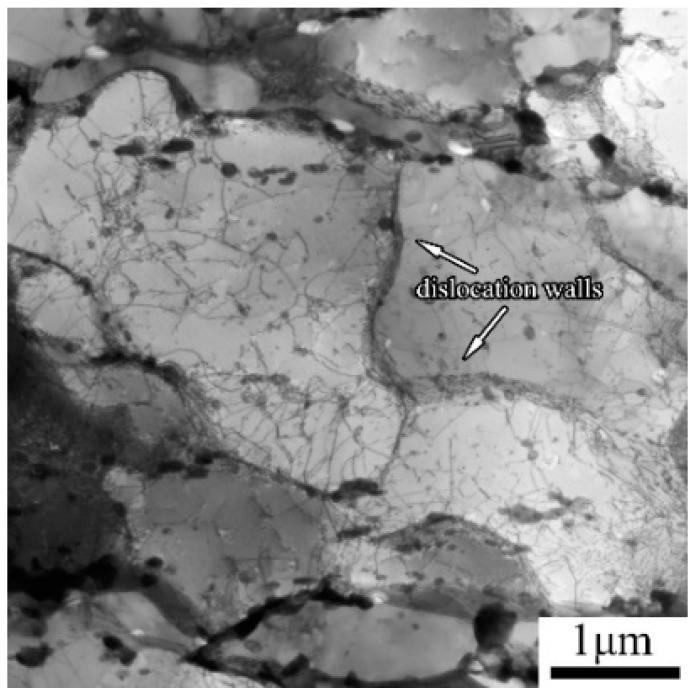
Dislocation walls in 9Cr-3W-3Co steel crept at 65 °C for 18,551 h.

**Figure 12:**
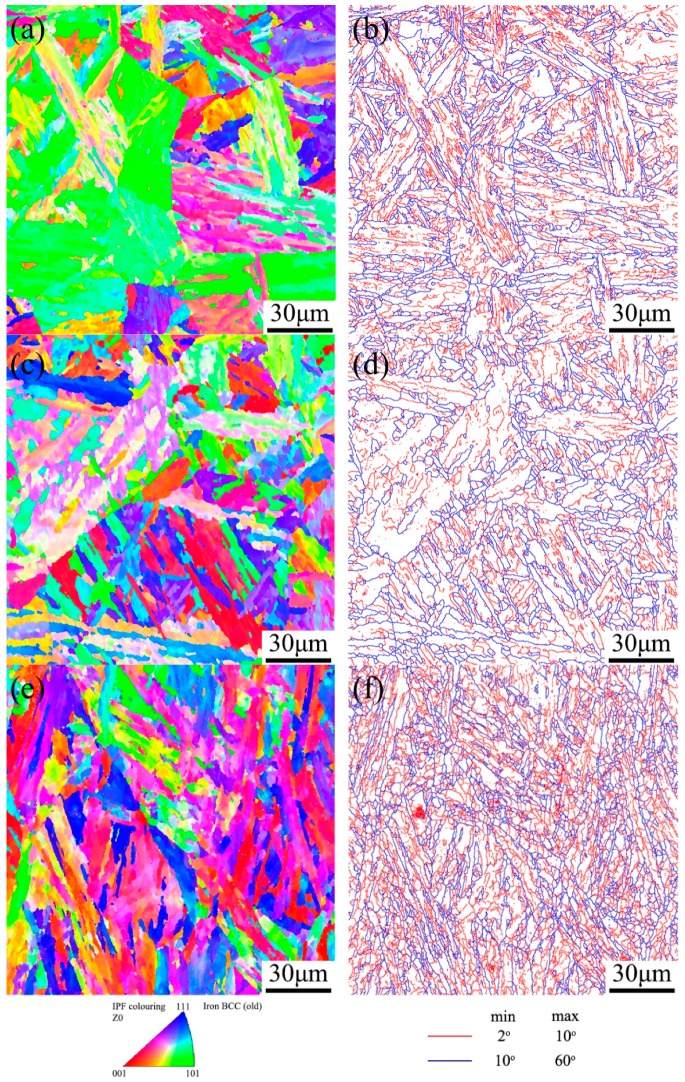
Electron back-scatted diffraction (EBSD) micrographs of 9Cr-3W-3Co steel crept at 650 °C for 18,551 h: (**a**,**c**,**e**) IPF figures, (**b**,**d**,**f**) boundary maps. (**a**,**b**) are from as-received specimen, (**c**,**d**) are from grip, (**e**,**f**) are from gauge.

**Figure 13:**
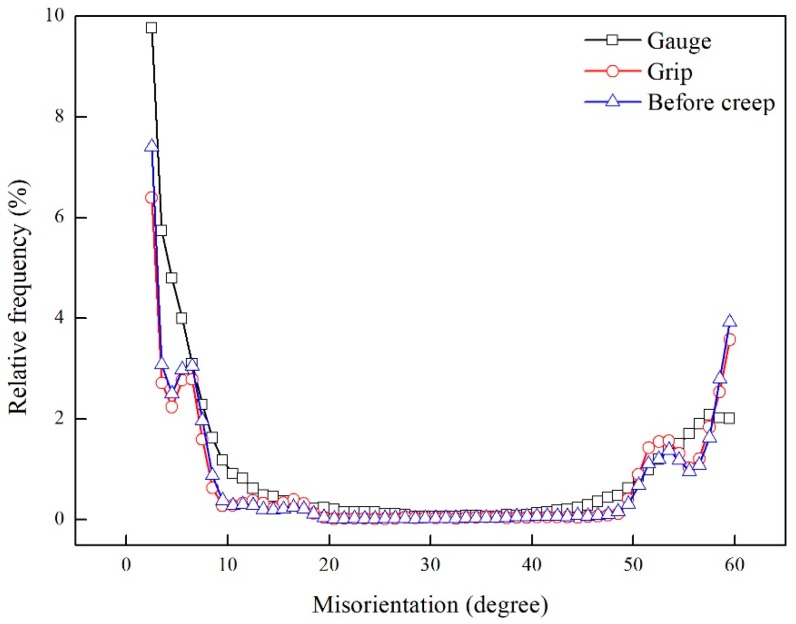
Misorientation angles for EBSD maps in Figure 12.

**Figure 14:**
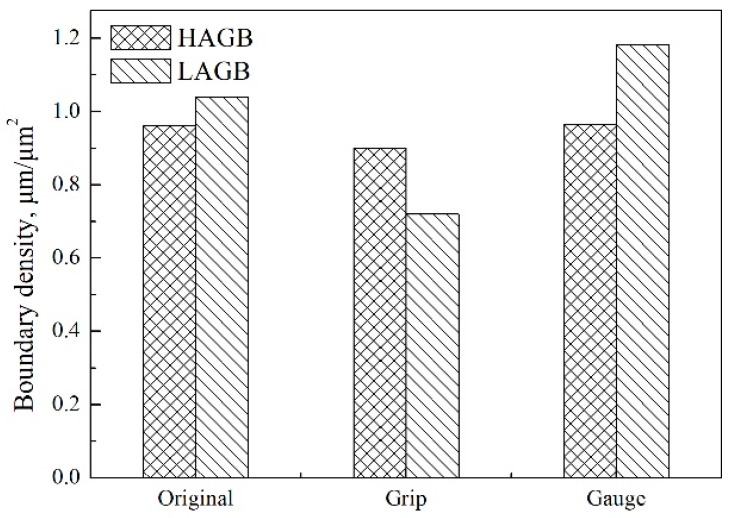
Boundary density of EBSD maps in Figure 11.

Except for the dislocations within martensite laths, some dislocations were generated near fractures during creep. The dislocations at high temperature atmosphere interacted rapidly and evolved into subgrain boundaries in a lamellar structure, as shown in Figure 11. Figure 12 demonstrates EBSD micrographs of samples before and after creep tests. The color in Figure 12a,c,e indicates the crystal orientations parallel to the normal direction of the figure, while the red and blue lines in Figure 12b,d,f suggest the HAGBs with a boundary misorientation of 10° or larger; and LAGBs with a boundary misorientation of 2° to 10°. For martensite steels, LAGBs contain boundaries between adjacent laths and subgrains, while HAGBs are block, packet and austenite grain boundaries. The variation in misorientation angle and boundary density relative to the as-received condition are given in Figure 13 and Figure 14. The result reveals a significant increase in the relative frequency and boundary density of LAGBs in the gauge area of long-term ruptured sample. Although some dislocations were partly annihilated, the strain-induced recovery brought new subgrain boundaries by the pile-up of dislocations (Figure 11), while new dislocations were continuously triggered by the stress. Moreover, a slight decrease of LAGBs in the grip area could also be seen, indicating that the reduction of dislocation in the stress-free grip area could not be effectively compensated.

Sanchez et al. [21] reported that many low angle boundaries could be seen in Grade.91 steel after long-term creep, which were characterized as equiaxed subgrains. Similarly, in the present study, a higher density of subgrains was generated at the stress-concentrated area and long aging up to 18,551 h had little effect on the distribution of misorientation. This might be the result of stabilized lath substructures of 9Cr-3W-3Co steels, as reported by Yan et al. [16]. Thus, the higher density of subgrains was the result of dynamic recovery, while the required activation energy for this process could be largely reduced by the stored energy from deformation.

### 4.2. Thermodynamic Calculation and Improvement of Creep-Rupture Strength

**Figure 15:**
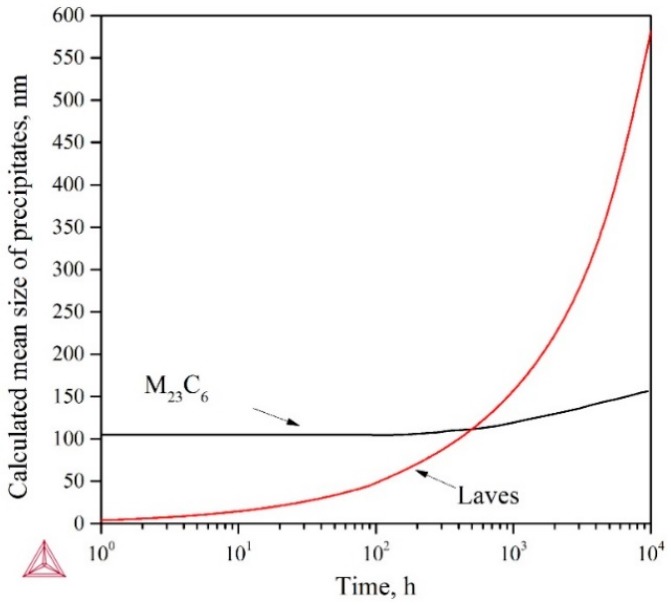
Precipitation kinetic of two precipitates in 9Cr-3W-3Co steel under 650 °C simulated by Thermo-Calc.

The pinning effect of second-phase particles on the grain and subgrain boundaries is one of the most significant strengthening mechanisms for 9–12% Cr creep-resistant steels. The migration of lath boundaries discussed in Section 4.1 indicates the weakened pinning effect after long-term creep, which is significantly affected by the average diameter of particles. In this study, the diameter of M_23_C_6_ and Laves were simulated using Thermo-Calc software, as shown in Figure 15. Given the high stability of MX phase in 9–12% Cr steel reported elsewhere [22,23], the simulation of MX was not performed in this case. Figure 15 indicates both precipitates grow gradually, while it is noticeable that the size of Laves phase is much larger than M_23_C_6_ after aging for 300 h or more.

Among all the precipitates, the Laves phase provides the highest pinning pressure at s applied stresses greater than 100 MPa, while the M_23_C_6_ carbides give the main contribution to the overall Zener drag force at an applied stress of 100 MPa [24]. Besides, it was found that the coarsened Laves phase particles initiated creep microcracks more easily than other precipitates [25,26,27]. Thus, the coarsening rate of Laves phase might be the critical factor that determines the creep rupture strength of 9Cr-3W-3Co steel. Therefore, higher microstructural stability of Laves phase might be beneficial to improve the creep rupture strength of this steel.

The main forming elements of Laves phase in 9Cr-3W-3Co steel consist of tungsten, iron and chromium, while the partition of chromium in Laves phase shows high stability during long-term service. Hence the tungsten content might be the most significant factor that determines the coarsening rate of Laves phase.

**Figure 16:**
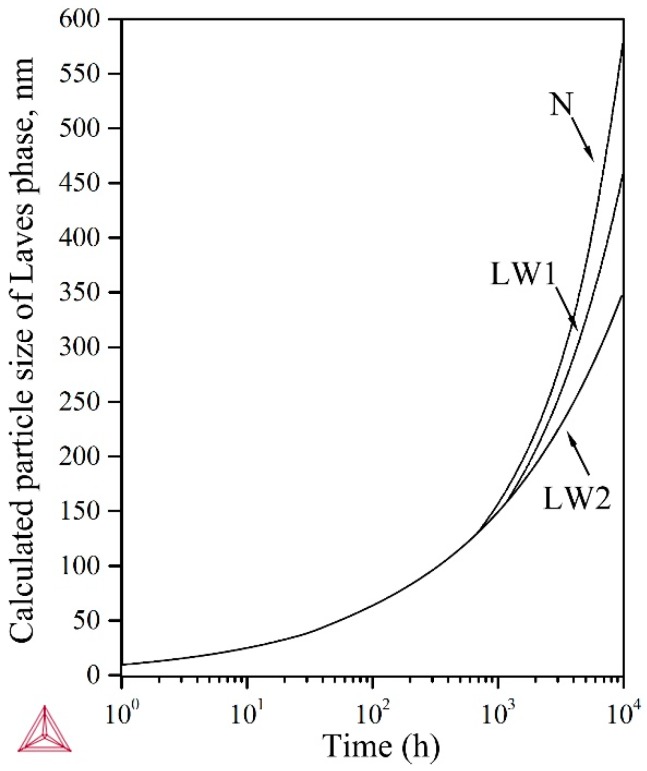
Effect of W content on precipitation kinetic of Laves phase in 9Cr3W3Co steel under 650 °C simulated by Thermo-Calc.

**Figure 17:**
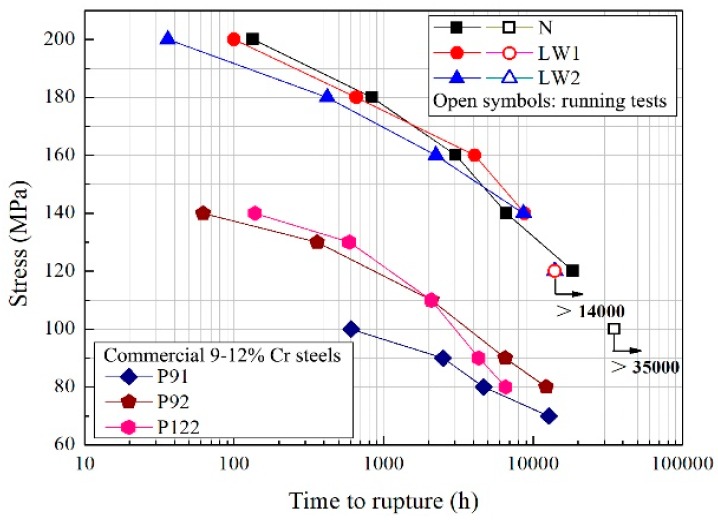
Creep rupture results of 9Cr-3W-3Co steels and commercial 9–12% Cr steels at 650 °C.

Apart from the original N# steel (2.96% W), two steels containing different tungsten concentration were investigated. Steels LW1# (2.63% W) and LW2# (2.32% W) were designed to reveal the effects of the variation of tungsten on the coarsening behavior of M_23_C_6_ carbides and Laves phase particles. The composition gradient of tungsten is around 0.3 pct. The thermodynamic simulation was carried out using Thermo-Calc. Figure 16 demonstrates the particle size of Laves phase evolved over time, indicating higher coarsening-resistance of Laves phase in steel containing less tungsten. These results exhibit the beneficial effect of less adding tungsten on weakening the coarsening behavior. Hence, it is reasonable to assume that sluggish coarsening behavior might lead to higher creep-rupture resistance. This speculation was well proved. The stress versus time to rupture curves of three 9Cr-3W-3Co steels at 650 °C are given in Figure 17. The results for three commercial 9–12% Cr steels, P91, P92 and P122, are also presented [28]. The three 9Cr-3W-3Co steels exhibited much better creep-rupture strength than the three commercial steels.

Table 2 compares the creep rupture time of three 9Cr-3W-3Co steels. Under stress of 160–200 MPa, the rupture time of LW1# and LW2# was lower than that of N#. However, LW1# and LW2# showed longer creep-rupture time than N# when stress was 140 MPa. Though the creep tests for LW1# and LW2# were still running, these two steels showed excellent creep-resistance. The tensile tests of specimens were also conducted at 650 °C. The results are given in Table 3. It can be seen that the yield and ultimate strengths of N# steels are slightly higher than those of the other two steels, indicating that higher W content has a beneficial influence on strength. For specimens under normalization and tempering, tungsten is mostly distributed in matrix, which greatly contributes to strength by a solid-solution effect. Steels containing more tungsten therefore show stronger solid-solution strengthening effect. Although higher W content is more favorable to tensile properties, a slight decrease of this alloying element in 9Cr-3W-3Co steel needs to receive more attention from the point of view of higher creep rupture property.

## 5. Conclusions

The profound influence of stress on precipitation evolution and martensite lath recovery of 9Cr-3W-3Co steel at 650 °C was investigated in detail. For specimens crept at stress higher than 140 MPa, a high density of dislocation was triggered, inducing precipitation of large number of second phase particles. However, a lower level of stress greatly accelerated the coarsening behavior of Laves phase particles and the transformation from lath substructure to large size subgrains. This mixed effect could be attributed to the interrelation of strain-induced precipitation and stress-induced recovery, while the coarsening rate is a dominant factor that affects the recovery of lath substructure. Thermodynamic simulation reveals that decreasing tungsten content of 9Cr-3W-3Co steel retards the coarsening behavior of M_23_C_6_ and Laves. This was further confirmed by creep test results of 9Cr-3W-3Co steels containing different tungsten concentrations. The optimization of tungsten content resulted in longer creep rupture time at 650 °C.

## Figures and Tables

**Table 1:** Chemical compositions of the steels studied (mass %).

Heat	C	Si	Mn	P	S	Cr	W	Co	V	Nb	N	B	Cu
N#	0.080	0.36	0.58	0.0025	0.0016	9.03	2.96	3.02	0.20	0.058	0.0049	0.015	0.90
LW2#	0.088	0.23	0.52	0.0023	0.0017	8.88	2.63	3.02	0.20	0.051	0.0088	0.015	0.91
LW1#	0.082	0.20	0.53	0.0027	0.0013	8.81	2.32	3.00	0.20	0.050	0.0097	0.012	1.03

**Table 2:** Creep rupture time of 9Cr-3W-3Co steels at 650 °C.

Stress, MPa	Creep Rupture Time, h		
	N	LW1	LW2
200	134	100	36
180	837	659	420
160	3037	4082	2243
140	6667	8791	8635
120	18,551	>14,000	>14,000
100	>35,000	–	–

**Table 3:** Tensile properties of three 9Cr-3W-3Co steels at 650 °C.

Tensile Property	N# (2.96% W)	LW1 (2.63% W)	LW2 (2.32% W)
Yield strength (R_p0.2_), MPa	366	335	340
Ultimate strength (R_m_), MPa	391	387	385
Area reduction (Z), %	27.5	28	35
